# Insight into genetic, biological, and environmental determinants of sexual-dimorphism in type 2 diabetes and glucose-related traits

**DOI:** 10.3389/fcvm.2022.964743

**Published:** 2022-11-24

**Authors:** Amel Lamri, Monica De Paoli, Russell De Souza, Geoff Werstuck, Sonia Anand, Marie Pigeyre

**Affiliations:** ^1^Department of Medicine, McMaster University, Hamilton, ON, Canada; ^2^Population Health Research Institute (PHRI), Hamilton, ON, Canada; ^3^Thrombosis and Atherosclerosis Research Institute (TaARI), Hamilton, ON, Canada; ^4^Department of Health Research Methods, Evidence, and Impact, McMaster University, Hamilton, ON, Canada

**Keywords:** genetics, type 2 diabetes, sex-specific effect, sex-dimorphism, gestational diabetes, genome wide association studies, animal models

## Abstract

There is growing evidence that sex and gender differences play an important role in risk and pathophysiology of type 2 diabetes (T2D). Men develop T2D earlier than women, even though there is more obesity in young women than men. This difference in T2D prevalence is attenuated after the menopause. However, not all women are equally protected against T2D before the menopause, and gestational diabetes represents an important risk factor for future T2D. Biological mechanisms underlying sex and gender differences on T2D physiopathology are not yet fully understood. Sex hormones affect behavior and biological changes, and can have implications on lifestyle; thus, both sex-specific environmental and biological risk factors interact within a complex network to explain the differences in T2D risk and physiopathology in men and women. In addition, lifetime hormone fluctuations and body changes due to reproductive factors are generally more dramatic in women than men (ovarian cycle, pregnancy, and menopause). Progress in genetic studies and rodent models have significantly advanced our understanding of the biological pathways involved in the physiopathology of T2D. However, evidence of the sex-specific effects on genetic factors involved in T2D is still limited, and this gap of knowledge is even more important when investigating sex-specific differences during the life course. In this narrative review, we will focus on the current state of knowledge on the sex-specific effects of genetic factors associated with T2D over a lifetime, as well as the biological effects of these different hormonal stages on T2D risk. We will also discuss how biological insights from rodent models complement the genetic insights into the sex-dimorphism effects on T2D. Finally, we will suggest future directions to cover the knowledge gaps.

## Introduction

Type 2 diabetes (T2D) is a metabolic disorder characterized by a combination of insulin resistance, (especially in adipose tissue, skeletal muscles and the liver), and relative insulin secretion deficiency (1). A wide variety of lifestyle factors, including excess body weight, smoking, and a sedentary lifestyle increase the risk of developing T2D (2). Globally, over 422 million individuals are affected by T2D worldwide and over 1.5 million deaths are annually attributed to T2D, ranking it among the top ten leading causes of mortality (3). Notably, epidemiological studies show that men develop insulin resistance and T2D earlier than women and at a lower BMI (4, 5). Premenopausal women have a reduced risk of developing T2D, compared to men or postmenopausal women (4, 6), but when women reach menopause, the risk becomes similar to that of men (7, 8).

Sex and gender differences play an important role in pathophysiology of T2D (4). Sex differences refer to biological differences, which are caused by differences in sex chromosomes, sex-specific gene expression, sex hormones, and their effects on organ systems. Gender differences refer to the effect of identities, expressions and societal roles and their implications on lifestyles. Both sex- and gender-specific biological and behavioral risk factors interact within a complex network to explain the differences in T2D risk and physiopathology in men and women. Polycystic ovary syndrome, gestational diabetes mellitus (GDM), and the age of menopause are three major woman-specific risk factors of T2D. For instance, women diagnosed with polycystic ovary syndrome typically have significant insulin resistance, regardless of body weight (9). Of women who had a history of GDM and were not given metformin or provided lifestyle interventions, almost 50% developed T2D within 10 years (10). T2D risk increases for women entering menopause before the age of 40, with a 30% increase in risk of T2D vs. women entering menopause from 50 to 54 years old (11). Moreover, women with T2D face an increased risk for cardiovascular disease that is at least two to four fold higher than the increase in cardiovascular disease risk seen in men with T2D (12). Premenopausal women without T2D are at a lower risk for cardiovascular disease than men without T2D of the same age, and much of this protection from cardiometabolic risk is thought to be due to the effects of estrogen, including estrogen receptor-mediated effects on lipid and glucose metabolism, endothelial function, and fat deposition (13). Estrogen appears to be cardioprotective unless T2D is present, but after the menopause, the protective effects are lost as estrogen deficiency develops (12).

From a biological perspective, T2D pathophysiology is partially driven by genetic factors (14). Thereby, progress in genetic studies have facilitated the identification of more than 300 loci associated with glucose-related variables and T2D. These studies have led to a better understanding of the biological pathways involved, as well as the common underlying biological mechanisms linking polycystic ovary syndrome and T2D (15), or GDM and T2D (16). Several rodent models of diabetes exhibiting an exaggerated form of sexual dimorphism have also been useful to delineate the mechanisms underlying the protection against T2D conferred by estrogens.

In this narrative review, we will focus on the current state of knowledge on the sex-specific effects of genetic factors associated with glucose-related traits, insulin resistance and T2D during the life course. Given that sex hormones dramatically change during a women's lifetime (e.g., ovarian cycle, pregnancy, and menopause), we will discuss the biological effects of these different hormonal stages on type 2 diabetes risk. We will further discuss how rodent models provide evidence on the effects of sex-dimorphism on type 2 diabetes risk. We will finally suggest future directions to cover the current knowledge gaps.

## Genetic evidence for sexual dimorphism in type 2 diabetes

Regulatory differences in blood glucose and insulin levels are both heritable traits. When estimated separately, the single nucleotide polymorphism (SNP)-based heritability (17) for T2D is significantly higher in men than in women (18), which suggests differences in the underlying genetic determinants of T2D risk due to sex-dimorphic effects in specific genes (or loci).

Given the complex nature of T2D related traits, Genome wide association studies (GWAS) have been one of the most powerful approaches used to identify new loci. While the methodology has essentially remained unchanged since the publication of the first T2D GWAS (19), the list of associated genes has considerably expanded due to the increase in sample size, and the inclusion of samples of multiple ancestries (20, 21). However, the proportion of variance in glucose related traits is still not fully explained by the currently known loci, and gene × gene and gene × environment interactions are thought to contribute to this missing heritability, with gene × sex interactions being one of these modulators. Three main statistical approaches have been used to date to investigate sex-dimorphic effects. The first approach involves performing sex-stratified analysis, followed by a heterogeneity test of effect between the two sexes at each genetic locus. The second approach consists in performing a single analysis with sex and SNP × sex interaction terms in the statistical model tested (22). The third, referred to as a sex-differentiated test, combines data for both sexes in a single analysis, includes the number of X-chromosome copies (1 or 2) as an independent variable in the model and allows for testing heterogeneity of allelic effects between males and females at the cost of one degree of freedom (22). Each one of these approaches have their own limitations as to the ability and power to detect sex-dimorphic effects (19, 20). However, one major advantage of the first approach is the possibility of testing for sex-related heterogeneity using summary statistics of publicly available sex-stratified data, while the other two methods require individual level data.

The spectrum of sex-dimorphic effects varies from (i) different (significant) direction of effects between males and females, (ii) similar (significant) direction of effects with differences in magnitude of the effect between sexes (e.g., larger effect sizes in one of the two sexes), to (iii) sex-specific effects whereby the association is significantly observed in one of the sexes only; this last case scenario being an extreme example of the second. It is important to note that the distinction between these three cases heavily relies on the statistical method, power, and parameters used to determine the significance of a SNP-T2D association in each sex. Hence, intermediary/inconclusive scenarios that do not clearly fit in the above-mentioned categories can often be observed (e.g., a SNP with a GWAS significant association (*p-value* < 5 x 10^−8^) in one sex and suggestive association only (*p-value* < 1 × 10^−6^) in the other. Therefore, balanced sample sizes in the two sex groups (and hence a comparable statistical power) is a crucial component for sex-comparison analyses.

## Human genes/loci with sex-dimorphic effects on glucose related-traits

Primary evidence of sex-specific effects in glucose metabolism-related genes/loci are rare, and replication attempts are even more sparse, due to the lack of power concomitant to the sex-stratified or the sex-interaction analyses. A large proportion of genes contributing to sex-dimorphic effects on FG and FI were described in a recent study lead by Lagou et al. (23). The authors conducted genome wide association studies (GWAS) in over 140,000 (for FG) and 98,000 (for FI) adult normoglycemic men and women (separately) of European ancestry from the Meta-Analyses of Glucose and Insulin-related traits Consortium (MAGIC). GWAS results were subsequently meta-analyzed and the heterogeneity of allelic effects between the two sexes was estimated (23). A targeted analysis for 36 and 19 previously established FG and FI loci that aimed to detect sex-dimorphic effects in these loci, was also conducted. To date, this study is largest one to investigate the modulating effect of sex on the genetic determinants of glucose-related traits on a genome wide level, complemented with genetic correlations and gene expression analysis. The absence of replication data represents the main limitations of this study.

Given the lack of replication studies and the weak significance of the associations in most studies, we investigated the sex-dimorphic effects of the autosomal genes/loci described in the literature with glucose-related traits [e.g., fasting glucose (FG), fasting insulin (FI), T2D], other correlated anthropometric traits including body mass index (BMI), waist to hip ratio (WHR), and GDM, using publicly available data from large genetic consortia, in order to identify the candidate genes with the most robust evidence of sex-dimorphic effect (spanning across multiple phenotypes). The list and description of genes/loci with the strongest evidence of sexual dimorphism across multiple traits is presented in Table 1. Results of an assessment of the sex-specific effects for each gene/locus on anthropometric and glucose-related traits is presented in Tables 1, 2.

**Table 1:** Associations of sex dimorphic effects of most important genes identified to date on type 2 diabetes and related phenotypes.

**Gene/locus**	**Reported lead SNP with sex-dimorphism**	**Effect /other allele**	**Primary phenotype**	**Direction of association for primary phenotype**	**Secondary phenotypes tested**	**Direction for secondary phenotype**	**# other SNPs with nominal sex heterogeneity**	**r^2^ range with reported lead SNP**
*COBLL1/GRB14*	*rs10195252*	T/C	FI	**Association with FI in women only** Women: Beta = 0.02, SE = 0.004, *P* = 1.23 × 10^−6^ Men: Beta = 0.007, SE = 0.004, *P* = 0.07 Sex heterogeneity (Cochran's Q-test) *P* = 0.03	BMI	**T allele associated with lower BMI in women** Women: Beta = −0.01, SE = 0.002, *P* = 9.58 × 10^−10^ Men: Beta = 0.006, SE = 0.002, *P* = 0.01 Sex heterogeneity (Cochran's Q-test) *P* = 0.02	32	0–1.00
					T2D	**T allele associated with higher T2D in women** Women: Beta = 0.08, SE = 0.01, *P* = 4.20 × 10^−15^ Men: Beta = 0.05, SE = 0.009, *P* = 4 × 10^−8^ Sex heterogeneity (Cochran's Q-test) *P* = 0.02	77	0–1.00
					WHR	**T allele associated with higher WHR in women** Women: Beta = 0.06, SE = 0.002, *P* = 6.35 × 10^−149^ Men: Beta = −0.005, SE = 0.003, *P* = 0.05 Sex heterogeneity (Cochran's Q-test) *P* = 1.81 × 10^−78^	120	0.4–1
					FG	NS-Cochran's Q-test *P* = 0.59	7	0–0.28
*ADCY5*	*rs11708067*	A/G	FG	**Stronger effect on FG in women** Women: Beta = 0.03, SE = 0.003, *P* = 5.01 x 10^−16^ Men: Beta = 0.02, SE = 0.004, *P* = 2.19 x 10^−6^ Sex heterogeneity (Cochran's Q-test) *P* = 0.04	BMI	NS-Cochran's Q-test *P* = 0.18	0	NA
					T2D	NS-Cochran's Q-test *P* = 0.28	21	0.17–0.21
					WHR	NS-Cochran's Q-test *P* = 0.69	14	0.04–0.27
					FI	NS-Cochran's Q-test *P* = 0.52	25	0.17–0.18
*PROX1*	*rs340874*	C/T	FG	**Stronger effect on FG in women** Women: Beta = 0.02, SE = 0.003, *P* = 1.69 × 10^−13^ Men: Beta = 0.01, SE = 0.003, *P* = 4.81 × 10^−4^ Sex heterogeneity (Cochran's Q-test) *P* = 0.01	BMI	NS-Cochran's Q-test *P* = 0.36	2	0.03–0.11
					T2D	NS-Cochran's Q-test *P* = 0.28	9	0.02–0.04
					WHR	**C allele associated with higher WHR in women** Women: Beta = 0.009, SE = 0.002, *P* = 0.0001 Men: Beta = −0.0001, SE = 0.002, *P* = 0.97 Sex heterogeneity (Cochran's Q-test) *P* = 0.009	9	0.09–0.65
					FI	NS-Cochran's Q-test *P* = 0.63	1	0.1
*RGS17*	*rs1281962*	C/G	FG	**Stronger effect on FG in women** Women: Beta = 0.01, SE = 0.003, *P* = 2.60 × 10^−7^ Men: Beta = 0.006, SE = 0.003, *P* = 0.04 Sex heterogeneity (Cochran's Q-test) *P* = 0.04	BMI	NS-Cochran's Q-test *P* = 0.33	7	0.00–0.11
					T2D	NS-Cochran's Q-test *P* = 0.12	103	0.00–0.37
					WHR	NS-Cochran's Q-test *P* = 0.85	19	0.05–0.42
					FI	NS-Cochran's Q-test *P* = 0.71	0	NA

Beta, mean change in primary phenotype per risk allele; SE, standard error for Beta; P, p-value of association between primary phenotype and risk allele; NA, not applicable; NS, not significant; #, number; r^2^, Linkage disequilibrium between SNPs.

**Table 2:** Summary of cross-phenotype sex-heterogeneity tests in BMI, WHR, T2D, FG, and FI consortium data at genetic loci with previously known sex-dimorphism.

**Gene/locus**	**Phenotype with known sex-dimorphism**	**New phenotype tested for heterogeneity**	**# SNPs tested in gene/locus**	# SNPs with *P* < 5 × 10^−6*^	# SNPs with *P* < 5 × 10^−6*^and P_Het < 0.05_	# SNPs with *P* < 5 × 10^−6*^and P_Het_ < P_Het Bonferroni_
*ADCY5*	Fasting glucose	Type 2 Diabetes	750	60	3	0
		WHR	98	9	9	4
		Fasting insulin	92	0	-	-
		BMI	92	0	-	-
*COBLL1/GRB14*	Fasting insulin	BMI	168	30	22	0
		Fasting glucose	103	0	-	-
		Type 2 Diabetes	922	51	41	0
		WHR	154	105	105	98
*RGS17*	Fasting glucose	Fasting insulin	109	0	-	-
		Type 2 Diabetes	631	124	88	0
		WHR	145	3	2	2
		BMI	154	0	-	-
*DSCAM*	Fasting glucose	BMI	980	13	1	0
		WHR	863	0	-	-
		Fasting insulin	440	0	-	-
		Type 2 Diabetes	5,542	0	-	-
*PROX1*	Fasting glucose	BMI	30	0	-	-
		WHR	17	6	6	2
		Fasting insulin	6	0	-	-
		Type 2 Diabetes	218	0	-	-

^*#^, number of SNPs with a suggestive association (P < 5 x 10^−6^) with the phenotypes in males only, females only, or both. A total of 22 autosomal loci (Table 3) were tested for association in 5 different consortia, for a phenotype other than the one for which the sex-dimorphism was originally described. This table only includes only loci with at least 1 SNP with P < 5 × 10^−6*^ and P_*Het*_ < 0.05 in at least one of the test consortia (BMI, WHR, FG, FI or T2D).

### Genes identified in childbearing women

#### Autosomal genes

##### *COBLL1/GRB14* locus

The locus which includes *growth factor receptor bound protein 14* (*GRB14*) and *cordon-bleu WH2 repeat protein like 1* (*COBLL1)* genes, has the strongest evidence of sexual dimorphism across multiple traits. *GRB14* encodes for a protein that binds to insulin, inhibiting its signaling activity (24). The biological function of *COBLL1*'s protein is unclear beyond its possible involvement in the Wnt/PCP pathway regulation in mammals (that regulates crucial aspects of cell fate determination, cell migration, cell polarity, neural patterning and organogenesis during embryonic development) (25), and its association to multiple metabolic traits and tumor types. SNPs in this gene displayed nominal evidence of sex-differences, with an association with FI observed in females only (23). The FI increasing allele of the lead SNP described by Lagou et al. (23) (*rs10195252*) was also significantly associated with a lower BMI and WHR in women only (effect of the same SNP in men was weaker and nominal) (Table 1). The same FI increasing allele (T) was associated with an increased risk of T2D in a female-specific manner (Table 1). Sex-differences influencing WHR (23–25), triglycerides (26) and T2D (27) at this locus have also been described in the literature. Finally, gene expression of *COBLL1* was higher in women's gluteal fat, abdominal fat and whole blood, while men had a higher expression than women in the liver (23). Levels of *GRB14* were nominally higher in women's gluteal fat. Levels of *GRB14* in abdominal fat were similar between men and women (23). Given all these results, it is possible that an effect of variants within/near to *COBLL1* and *GRB14* could influence glucose metabolism *via* adipose tissue and body fat distribution differences between men and women. More studies are needed in order to establish this link.

##### *ADCY5* gene

In sex-combined studies, SNPs in the *adenylate cyclase 5 (ADCY5*) gene are associated with multiple T2D-related traits including FG (23, 28–32), FI (21), glycated hemoglobin (33), HOMA-B (i.e., index of insulin secretion) and T2D (34–43), anthropometric and body fat distribution traits, such as WHR (44, 45), body fat percentage (46), BMI (47), inflammation phenotypes such as C-reactive protein (48), early life factors including gestation duration (49), birth weight (50–56), blood lipid levels, including total cholesterol (57, 58), HDL-cholesterol, apolipoprotein A1 (59), and blood pressure measures (60). SNPs in the vicinity of *ADCY5* showed sex-dimorphic effects in association to FG. The lead SNP associated with FG described by Lagou et al. (23) (*rs11708067*) did not show evidence for sexual dimorphism when tested for FI, T2D, WHR or BMI (Table 1). Nevertheless, another variant in this gene likely independent from the aforementioned SNP (*rs3934729*), shows nominal sex-dimorphic effect with a stronger effect in women (Table 1). The sex-dimorphic effect of *rs11708067* on T2D risk was also previously described in the literature (27). In a gene expression analysis, *ADCY5* SNPs were associated with levels of sex hormone binding protein (SHBG) (61), which provides a possible clue as to the mechanisms and pathways involved in *ADCY5's* sex-specific effects.

##### *PROX1* gene

The *prospero homeobox 1* (*PROX1*), which encodes for the Prospero homeobox protein 1 transcription factor plays a key role in embryonic cellular development and differentiation in multiple organs of complex organisms including *Drosophila*, mice and humans (62, 63). On a molecular level, *PROX1* was mostly studied for its prominent role in lymphatic endothelial cell fate determination of which it is generally considered as the master regulator (64). Mutations in *PROX1* have been associated with multiple forms of cancer. In human adult GWASs, *PROX1* genetic variants have been overwhelmingly associated with glycemic traits overall (65), blood glucose levels (23, 30, 31, 66, 67), glycated hemoglobin levels, HOMA-B, T2D (32, 34–37, 39, 43), and to a lesser extent to birth weight (51), and other cardiometabolic traits such as triglycerides (68) or WHR (suggestive association only) (45). As an established FG locus, Lagou et al. (23) tested and observed nominal evidence for sex heterogeneity in multiple *PROX1* genetic variants on FG, where the associations were stronger in women. This potentially sex-dimorphic FG SNP described by Lagou et al. (23) (*rs340874*) did not show evidence for association with BMI but nominal association with FI in both sexes, with no significant sex-related heterogeneity. However, the FG increasing allele (T) was associated with higher WHR in women only, with a significant heterogeneity. Multiple other SNPs located next to this locus showed nominal sex-dimorphic effects on WHR (Table 1). Possible clues as to how *PROX1* could influence glycemic traits in a heterogenous manner between men and women come from other GWASs. Indeed, SNPs in the vicinity of *PROX1* and its neighboring non-protein coding *PROX1 Antisense RNA 1 (PROX1-AS1)* genes have been shown to be associated with SHBG levels in both men and women (61, 69). However, the sex-dimorphic SNP in *PROX1* (*rs1281962*) described by Lagou et al. (23) was not associated with SHBG levels and more investigations are required in order to establish a connection between sexual dimorphism for SNPs near *PROX1*, SHBG, and FG. *PROX1* SNPs were also associated with testosterone levels at a GWAS-significance level in men, but not in women, which could suggest a sex-dimorphic effect (61). Whether the *PROX1* plays a differential role in glucose related traits through its regulation of SHBG and testosterone levels remains to be determined.

##### *RGS17* gene

The *regulator of G protein signaling 17 (RGS17)* gene and its corresponding RGS17 protein are involved in the regulation of multiple G protein-coupled receptor signaling cascades (70). SNPs in *RGS17* displayed strong associations with HDL-cholesterol (32, 57–59), triglycerides, apolipoprotein A1, FG (21, 23, 31), glycated hemoglobin, T2D (34), WHR (45), BMI (39, 44, 47, 71, 72), diastolic blood pressure (60), and C-reactive protein. Multiple SNPs in the *RGS17* gene revealed larger effects on FG in women at nominal significance (23). The FG-increasing allele in the lead SNP was associated with higher BMI in a GWAS meta-analysis with larger effects in women (45). The lead SNP (*rs1281962*) did not show evidence for sexual dimorphism on BMI, T2D, FI or WHR (Table 1). However, two other SNPs in *RGS17* gene (*rs3910736, rs514784*) showed a weak association with WHR in women only (Table 1). However, these sex-dimorphic effects were not significant after correction for multiple hypothesis testing. No significant interaction was detected at this locus (72). A nominally significant heterogeneity effect was observed against T2D (Table 3). Given its pleiotropic role on cardio-metabolic traits, the mechanisms by which *RGS17* might differentially affect phenotypes remains unclear.

**Table 3:** Genes/loci with suggestive evidence of sexual dimorphism on type 2 diabetes and related phenotypes.

**Gene/locus**	**Chr**	**Phenotype with evidence of sex-dimorphism**	**Stronger sex-specific effect**	**Other evidence of sex-dimorphism**
**European populations**				
*IRS1*	2	FI (23)	Men	WHR (72), body fat percentage
*COBLL1/GRB14*	2	FI (23, 72)	Women	WHR (72), TG (26, 73–75)
*PROX1*	1	FG (23)	Women	
*ADCY5*	3	FG (23)	Women	
*PCK1*	20	FG (23)	Women	
*SLC30A8*	8	FG (23)	Women	
*COL26A1 (EMID2)*	7	FG (76)	Women	
*ZNF12*	7	FI (23)	Women	
*RGS17*	6	FG (23)	Women	BMI (45)
*HKDC1*	10	GDM (16)	Women	
*MC4R*	18	T2D (77)	Women	
*DGKB*	7	T2D (27)	Men	
*BCAR1*	16	T2D (27)	Men	
*KCNQ1*	11	T2D (27)	Men	
*CCND2*	12	T2D (27)	Men	
*MTNR1B*	11	GDM (16), 2-h plasma glucose (78)	Women	
*BCL11A*	2	T2D (27)	Men	
*BCLAF3/MAP7D2*	X	T2D (34)	Men	
*SPIN2A/FAAH2*	X	T2D (34)	Men	
*AR/OPHN1*	X	T2D (34)	Men	
*CCNQ/DUSP9*	X	T2D (34)	Men	
*BACE2*	21	fasting C-peptide (pregnancy) (78)	Women	
*NKX2-6*	8	FI, FG (72)	Women	WHR (72)
*LY86*	6	FG (72)	Women	WHR (72)
*EYA1*	8	T2D, FG (72)	Women	WHR (72)
*KLF14*	7	T2D (27), FI (72)	Women	WHR (72)
*NMU*	4	FI (72)	Women	WHR (72)
*PIGU*	20	HOMA-B, HOMA-IR (72)	Women	WHR (72)
*IQGAP2*	5	FI (72)	Men	WHR (72)
**Non- European populations**				
*DSCAM*	21	FG (79)	Women (Koreans)	
*SIRT1*	10	T2D (80)	Women (Pima Indians)	

#### Genes on chromosome X

Only few GWASs of glucose- and T2D-related traits have analyzed and identified chromosome X genetic variants (32, 34, 43, 81–83). This is partially due to the difference in the number of copies of sex chromosomes between men and women, which requires them to be analyzed separately from the autosomes. However, given the male heterogamety nature of sex determination in humans, and that the genes coding for some major regulators of sex steroids levels are located on this chromosome [e.g., *sex hormone binding globulin (SHBG) and androgen receptor (AR)*], loci on Chr X are candidates for gene × sex interactions. Among the studies that identified and validated the largest number of chromosome X variants directly associated with T2D, there was an analysis lead by Vujkovic et al. (34). This study included participants from multiple ancestries and identified a total of ten T2D loci on chromosome X (34). Among these, SNPs at the *AR/OPHN1* locus which displayed male-specific effects (non-significant in females) on T2D risk (Table 3). *AR* is an interesting biological candidate since this codes for the steroid hormone and transcription factor androgen receptor implicated in the expression of multiple male sexual development and differentiation genes under the control of testosterone (84). SNPs in *AR* have been associated with fasting insulin levels, and with baldness in males (34). The mechanism by which the *AR* gene indirectly influences results in sex-dimorphic phenotypes in relation to glucose metabolism still needs to be investigated.

### Genes identified during pregnancy

Given the female-specific aspect of pregnancies, genes associated with GDM during the unique physiological state of pregnancy can be considered as sex-dimorphic. GDM is thought to be closely related to T2D from a genetic perspective. T2D polygenic risk score has been strongly associated with risk of GDM, although only a few GDM GWASs have been performed (16, 78, 85). The following genes have been identified.

#### *HKDC1* gene

Although the majority of GDM associated genetic loci were previously known for their association with T2D or glycemic traits (86, 87), two GWASs reported *hexokinase domain containing 1 (HKDC1)* SNPs to be associated with GDM (78), and 2h-post load glucose test (16). *HKDC1* also appears to influence other early life factors, including maternal genetic effect on birth weight (51). This locus was not known for association with glycemic traits or T2D in non-gravid populations except for glycated hemoglobin (31). However, SNPs in *HKDC1* were significantly associated with SHBG levels in sex-combined and women only groups, but not in males (61). Other traits associated with *HKDC1* are mostly related to multiple blood cell count phenotypes (32, 88–91) and liver function [e.g., alanine aminotransferase (31, 92, 93), and aspartate aminotransferase].

#### *MTNR1B* gene

The *melatonin receptor 1B* (*MTNR1B*) gene is a well-established T2D locus with significant associations with multiple glucose-related traits including FG (21, 23, 28–30, 67, 94–101), glycated hemoglobin (31–33, 102, 103), insulin levels, insulin disposition index and insulin secretion rates (104, 105), acute insulin response, HOMA-B (21), and T2D (27, 34, 35, 37–40, 42, 43, 106, 107). The gene is also associated with various sleep and circadian rhythm-related phenotypes (108, 109). Given the strong association with T2D, it is not surprising that this gene has also been associated with GDM in several GWASs (16, 110). Despite the absence of evidence of sex-dimorphic effects in *MTNR1B*, this locus is of particular interest given that the effect size of *MTNR1B* SNPs are higher in GDM than T2D (16), which suggests a female-specific effect of this locus during pregnancy.

## Insights from genetic studies in rodents

The use of rodent models in the study of human disease is an important component of translational knowledge. It has the dual function of either confirming what was observed in human genetic studies or provide more information on the mechanisms by which specific genes can be associated with the development of T2D. Although wild type rodent models do not spontaneously develop diabetes, the condition can be induced genetically or chemically. Examples of human genes whose functional implication in T2D has been validated in mice are numerous. For instance, a human GWAS analysis showed that *SLC30A8's* SNPs are associated with susceptibility to T2D. Subsequent studies showed that the deletion its mouse homolog *Slc30a8* induced defects in insulin secretion and an overall impairment in glucose homeostasis (111).

The implication of genetics factors in diabetes-related sex-dimorphic effects in rodents is evidenced by the fact that different strains, with different genetic backgrounds, display different sex-dimorphic phenotypes in otherwise similar environments. The characteristics of the different models that display sex-dimorphism phenotypes are provided in Table 4. Models with monogenic forms of diabetes provide direct evidence of the involvement of a gene in these sex-dimorphic outcomes. A clear illustration of this is the Zucker Diabetic Fatty (ZDF) rats, a widely used model of obesity caused by the mutation of the *leptin receptor* gene (*Lepr*, also known as *Fa*) (123). Male ZDF develop hyperglycemia, hyperinsulinemia, impaired glucose tolerance, while females are normoglycemic (124) (Table 4). This highlights the differential involvement of the *Lepr* gene and the Leptin/Melanocortin pathway in the development of diabetes between males and females. Another interesting rodent model is the aromatase-knockout (Arko) mice, which result from a targeted disruption of *Cyp19A1*, a gene that encodes for the aromatase, an enzyme involved in the production of endogenous estrogen (113). *Cyp19A1* knock down mice display a range of sex-dimorphic phenotypes as a result. Among these, male ArKO mice show signs of insulin resistance and impaired glucose homeostasis, whereas females develop glucose intolerance, but not insulin resistance (113). The importance of estrogen and all actors involved in its metabolism will be discussed below.

**Table 4:** Genetically and chemically induced rodent models of T2D showing sexual dimorphism in glucose homeostasis.

**Rodent model**	**Methods to induce diabetes**	**Phenotype**	**Genetic background**
Zucker Diabetic Fatty (ZDF) rats	Genetically induced, monogenic	Males develop hyperglycemia, hyperinsulinemia, impaired glucose tolerance Females are normoglycemic	Mutation (*fa/fa*) in the leptin hormone receptor (112)
ArKO mice	Genetically induced, monogenic	Males are insulin resistant and have impaired glucose homeostasis. Females are glucose intolerant, but not insulin resistant	Mice lack the *Cyp19A1* gene which encodes aromatase, an enzyme involved in producing endogenous estrogen (113)
Otsuka Long-Evans Tokushima Fatty (OLETF) rats	Genetically induced, polygenic	Males develop late onset diabetes Females are normoglycemic	Development of hyperglycemia is associated with three loci (*Dmo1, Dmo2, Dmo3*) situated in Chromosomes 1, 7, 14, respectively (114). Additionally, another gene (*ODB-1*) associated with the development of hyperglycemia is located in the X-chromosome (115)
TALLYHO/JngJ mice	Genetically induced, polygenic	Males are obese and develop insulin resistance and hyperglycemia Females are obese and normoglycemic	Hyperglycemia in the *TALLYHO/JngJ* mice is associated with a recessive non-insulin dependent diabetes mellitus locus, *Tanidd1*, situated in chromosome 19. This locus can interact with other loci such as *Tanidd2* (chromosome 13), Tanidd3 (chromosome 15), TallyHo-associated fat pad, *Tafat* (chromosome 6), TallyHo-associated body weight, *Tabw1* (chromosome 7) (116–118)
New Zealand Obese (NZO) mice	Genetically induced, polygenic	Both males and females are obese and present with impaired glucose tolerance, however only males develop overt T2D	*Zfp69* is likely the gene involved in the susceptibility locus *Nidd/SJL* on Chromosome 4 and is associated with the development of severe hyperglycemia, hypoinsulinemia, as well as beta cell degeneration. Other genes involved in the development of the diabetic phenotype are *Pctp, Nob3* (119)
Streptozotocin-injected rodent (STZ), Alloxan-injected rodent	Chemically induced (STZ and alloxan have selective toxicity to pancreatic beta cells)	Female rodents require higher doses and/or more frequent injections of these chemicals to induce diabetes, compared to males (120–122)	Diabetes can be induced by STZ and alloxan on any rodent model

Rodent models of polygenic forms of diabetes also exist, with some models displaying more sex-dimorphic traits than others (Table 4). However, like human studies, the identification of the genes involved in sex-dimorphic phenotypes in these polygenic and more complex forms of diabetes is more difficult and has been poorly studied in rodents. However, given the recent advances in gene editing and other functional genomic tools, several new models have been specifically developed for the study the genetic causes of sex-dimorphic traits (124, 125). Among the most interesting candidates is a mouse for which the sex determining region of Chr Y (*Sry* locus) involved in male sex determination is transferred from chromosome Y to chromosome 3 in a male mouse, therefore detaching gonadal development from sex chromosomes (126). Male mice with this manipulation are then crossed with female mice carrying two X chromosomes. The resulting offspring can be an XY or XX carrying *Sry* in chromosome 3 (corresponding to a model where the effects of sex hormones are independent from the gonadal status), or XY and XX without this manipulation (126). In a recent study, these mice underwent gonadectomy and were subsequently supplemented with either estrogen, testosterone, or a blank control (126). It was observed that in these mice, estrogen and testosterone decrease the gonadal regulation of gene expression in the liver, whereas they enhance it in the adipose tissue. Furthermore, the effect of estrogen is more prominent than that of testosterone. It was also observed that sex chromosomes seem to regulate the expression of the *Hccs* gene, which is involved in the regulation of multiple metabolic pathways. Additionally, the study showed that genes affected by sex hormones in the adipose tissue are highly enriched with variants associated with cardiometabolic diseases and traits (126).

The study just described is an example of how rodent models can be used to decipher the mechanisms by which various sex-related components can individually affect gene expression at the basis of sexual dimorphism observed in glucose-related traits.

## Effects of sex hormones on glucose and T2D-related traits

### In humans

Although there is no study conducted in humans to investigate the interaction of circulating sex hormone levels with genetic factors on T2D risk, overall evidence shows that circulating levels of sex-hormones directly or indirectly modulate the effects of the genetic susceptibility for T2D, through different pathways and mechanisms. First, estrogen regulates body composition and fat distribution. In women, high estrogen level is usually associated with less ectopic fat deposition, more favorable lipid profile, and less insulin resistance than in men (6). Premenopausal women store fat primarily in gluteofemoral depots, which are considered benign or metabolically beneficial, whereas men tend to store fat in abdominal depots (6). In addition, estradiol has a beneficial impact to decrease visceral adipose tissue and increase brown adipose tissue (127). During female-specific age-related transitions, estrogen loss leads to decreased physical activity, increased adiposity with redistribution of fat to abdominal depots (128), and decreased muscle mass, whereas estrogen replacement reverses these changes (6). Second, excess of androgens and lower levels of SHBG have a direct effect on insulin resistance and T2D (129). Interestingly, sex hormones differentially modulate glycemic status and risk of T2D in men and women. High testosterone levels are associated with higher risk of T2D in women but with lower risk in men; the inverse association of SHBG with risk seems to be stronger in women than in men (130). Moreover, in women, low SHBG levels predict higher T2D risk, regardless of BMI and age (130). However, the causal relationships between low SHBG levels and T2D risk have been reported similarly in both sexes (131), though an effect on insulin resistance (132). Interestingly, lower estradiol level in men predicts lower risk of T2D (133).

Evidence on the effects of sex hormones on overall genetic susceptibility for T2D also comes from the usage of hormone replacing therapies (HRT) administered to prevent consequences of menopause including vasomotor/genitourinary symptoms and osteoporosis (134). Several clinical studies have demonstrated that HRT is beneficial for glucose homeostasis (135–138). Evidence provided by the North American Menopause Society/American College of Cardiology/American Heart Association, suggest that in women of < 60 y-old, within 10 years after menopause onset, menopausal hormone therapy with estrogens (combined or not with progestogen) may be beneficial for the prevention of coronary disease and may also reduce the incidence of T2D (139). In post-menopausal women with T2D, menopausal hormone therapy improves glycemic control, and insulin sensitivity (139), by improving β-cell insulin secretion and insulin sensitivity (137).

The study of transgender individuals also provides a unique opportunity to determine which metabolic functions are modulated by the prevailing milieu of sex steroids, because the chromosomal configuration remains unchanged (140). Usage of estrogen therapy in transgender women, as a feminizing hormonal therapy for individuals assigned male sex at birth, showed a significant effect on body composition within 12 months and these changes persist over the time, such as transgender women on estrogen lost lean mass and gained fat mass (141). Estrogen and antiandrogen therapy seemed to be associated with an increase in the absolute amount of visceral fat and subcutaneous fat, but a reduction in the ratio of visceral to subcutaneous fat (142). Effects on insulin sensitivity are controversial. Although transfeminine people may be at higher risk for T2D compared with cisgender women, the corresponding difference relative to cisgender men was not discernable in the STRONG cohort (143). There was little evidence that T2D occurrence in either transgender women or transgender men was attributable to gender-affirming hormone therapy use (143). Data from another large gender identity study suggested that both transgender men and women exhibited higher incidence of T2D than the general population (144), with a higher CV mortality rate among transwomen but not among transgender men. Despite receiving similar estrogen therapy, transgender women who elected orchiectomy had improved metabolic health compared with transgender women who retained their testes. Furthermore, data suggest that suppression of endogenous testosterone in transgender women appears to improve insulin sensitivity and reduce hepatic steatosis (145). Thus, the implications for long-term T2D incidence or cardiovascular health are still unclear, due to a paucity of long term prospective controlled studies. It should also be noted that most of the participants in these studies are of white European ethnicity, which limits the generalizability of the findings to transgender individuals of other ethnic groups.

### In rodents

A wide variety of interventions have been used to study the impact of hormonal changes on sex differences in the presence of T2D in rats and mice, with overall results that validate and strengthen the observations made from human studies. Both chemical and surgical approaches can be used to mimic menopause in any rodent model of choice (146). Chemically induced menopause can be attained by exposing rodents to the chemical 4-vinylcyclohexene diepoxide (VCD), which gradually depletes ovarian follicles and therefore mimics the perimenopausal and menopausal stages (147). It has been shown that the loss of ovarian function is associated with an increase in insulin resistance, the development of metabolic syndrome, and T2D (148). Interestingly, another study in VCD mice showed that hyperglycemia was significantly more severe in VCD female mice post-ovarian failure, compared to cycling females (149). Surgical menopause through ovariectomy, which induces immediate estrogen depletion, is the most used models to induce menopause in rodents. The detrimental effects of ovariectomy on glucose homeostasis have been analyzed in several mouse models of diabetes. For instance, female normoglycemic ZDF rats, showed impaired glucose homeostasis after ovariectomy (123). Ovariectomized female New Zealand obese (NZO) mice, who otherwise rarely develop diabetes (150), display severe hyperglycemia with a significantly higher prevalence compared to sham operated controls (151). Similar observations can be made in Wistar rats and C57BL/6J mice, two other rodent models that are typically normoglycemic, but where ovariectomy can impair their glucose homeostasis.

Estrogen supplementation, another commonly used chemical procedure in rodents, has been shown to restore glucose homoeostasis in ovariectomized female ZDF rats (123), and improve glucose tolerance and fasting blood glucose levels in males (152). Estrogen replacement reversed insulin resistance and visceral fat accumulation, and improved insulin sensitivity in the skeletal muscle (when combined with high fat diet) in ovariectomized female Wistar rats (153, 154). Estrogen supplementation in ovariectomized female C57BL/6J mice significantly improved blood glucose levels, glucose-stimulated insulin secretion as well as insulin content in pancreatic beta cells (155) and improved insulin sensitivity in the hepatic tissue (156). Finally, in the ArKO mice, estrogen replacement also significantly improved glucose tolerance and insulin sensitivity in both males and females, further indicating the protective role of estrogen in glucose homeostasis (113).

Of note, rodent studies show that the impact of sex hormones, combined to the exposure to environmental risk factors can lead to sexual dimorphic phenotypes as early as *in utero*. For example, in Sprague-Dawley rats, a multipurpose rodent model typically used in studies of metabolism and diabetes, male offspring born to mothers with diet-induced GDM had altered expression of genes associated with pancreatic growth, reduced beta cell expansion and differentiation, and impaired insulin secretion (157). Sex differences in glucose homeostasis of offspring born to C57BL/6J mice with diet-induced GDM have also been observed (158). Specifically, male offspring were more insulin resistant, had higher plasma insulin levels, and had smaller pancreatic islets compared to female offspring. Additionally, the expression of *PDX-1* which is involved in pancreatic beta cell maturation was significantly reduced in males, compared to female offspring. These data show that estradiol might exert protective effects in the metabolic homeostasis of female offspring.

Taken together, these studies illustrate how rodent models can help to recapitulate and pinpoint the mechanistic effects at the basis of sexual dimorphism as well as how they can be a valuable and complemental tool to inform genetic analysis on this specific area of research.

## Gender-specific genetic determinants for T2D risk

Gender-specific lifestyle behaviors including unhealthy diet, low physical activity and smoking are well-recognized risk factors for T2D that differ between gender (4), yet studies on interaction between gender-specific lifestyle behaviors and genetic determinants on T2D risk are still lacking, for the main reason that such studies need a very large sample size to reach the power required to detect significant effects. It has been shown that men are more likely to smoke than women (159, 160); women are more likely to consume a healthy diet (161, 162), but are less active than men [global average 32% for inactive women vs. 23% for inactive men (163, 164)]. Diets enriched in ultra-processed foods and sugar-sweetened beverages are associated with an increased risk of developing T2D (165), while diets enriched in fruits, whole grains, and dairy products are associated with a decreased risk of T2D (166). Moreover, the influence of lifestyle factors may exert their effects differently along the life stages.

## Knowledge gaps and conclusions

Most of GWASs studying the sex-dimorphic effects of genes on T2D risk have been conducted on populations of European ancestry. To date, the list of genes displaying sex dimorphic effects in non-European populations is extremely limited and includes *SIRT1*, identified in a population of Pima Indians (80), and *DSCAM* in Koreans (Table 3). However, the replication of sex-dimorphic effects at these loci is still needed, and larger, more diverse studies are still needed in order to identify more sex-dimorphic loci and increase our understanding on the interplay between genes, sex and the risk of diabetes.

**Figure 1:**
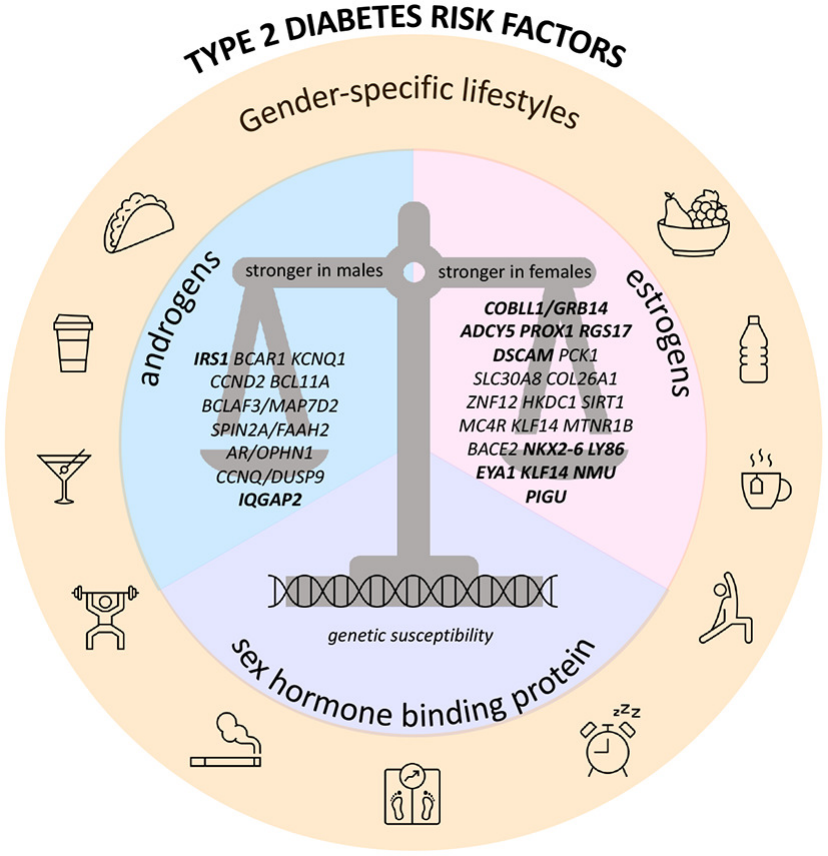
Recap figure depicting sex-specific genetic determinants of type 2 diabetes and glucose-related traits, and their interaction with endogenous/exogenous sex hormones, sex hormone binding protein, and gender-specific lifestyle factors. Hereby are presented the genes with sex-dimorphic effect on risk of T2D and/or GDM and/or FG and/or FI; genes in bold also have sex-dimorphic effect on BMI or WHR.

Sex and gender consideration in research studies has improved over the last 20 years, but preclinical research is still primarily done using male rodent models and male-derived cells, with the result that many conclusions are made based on incomplete and sex-biased data (167). Although sex-specific data can improve disease prevention, diagnosis, and treatment as well as reduce inequities, research to address the important goal of understanding key sex differences in cardiometabolic disease across the lifetime is still lacking. More studies are required to identify the mechanisms responsible for the sex-specific increase in cardiometabolic risk and to develop therapies that are safe and effective in women. Such research should take into account biological and behavioral factors that differ between women and men, including unique exposures in women, such as hormonal fluctuations across the life time from conception through aging (167) (Figure 1). Critical gaps and research priorities should include elucidation of the mechanisms whereby sex hormones regulate body composition, fat distribution and how they interact with diet and age, how are sex differences and race/ethnicity interrelated with T2D what are the mechanisms underlying the apparent paradox of effects of sex hormones in premenopausal women vs. postmenopausal women vs. men, what are the sex differences in therapeutic treatments and effects of T2D drugs, cardiovascular disease outcomes, and what are the metabolic impacts of hormonal replacement therapy, and of androgen and estrogen use in the context of biologically different sex. Previously published reviews on T2D genetics did not address the sex-specific differences on T2D genetic susceptibility. Our review is the first to synthesize the data on sex differences in relation to its effects with genetic variants on T2D and related traits. These offer a solid basis for future research in this field. Ultimately, expanding our understanding on why risk of T2D differs between men and women during life course could lead to the identification of new therapeutic targets to prevent T2D more effectively in both women and men.

## Author contributions

AL, MD, and MP wrote the manuscript. RD, GW, and SA provided guidance and edited the manuscript. All authors have approved the manuscript submission.

## Conflict of interest

The authors declare that the research was conducted in the absence of any commercial or financial relationships that could be construed as a potential conflict of interest.

## Publisher's note

All claims expressed in this article are solely those of the authors and do not necessarily represent those of their affiliated organizations, or those of the publisher, the editors and the reviewers. Any product that may be evaluated in this article, or claim that may be made by its manufacturer, is not guaranteed or endorsed by the publisher.
